# Supplementary figures and images for: Phage-inducible chromosomal islands promote genetic variability by blocking phage reproduction and protecting transductants from phage lysis
Source: PLoS Genet. 2022 Mar 28;18(3):e1010146. doi: 10.1371/journal.pgen.1010146 (PMC8989297; doi:10.1371/journal.pgen.1010146)

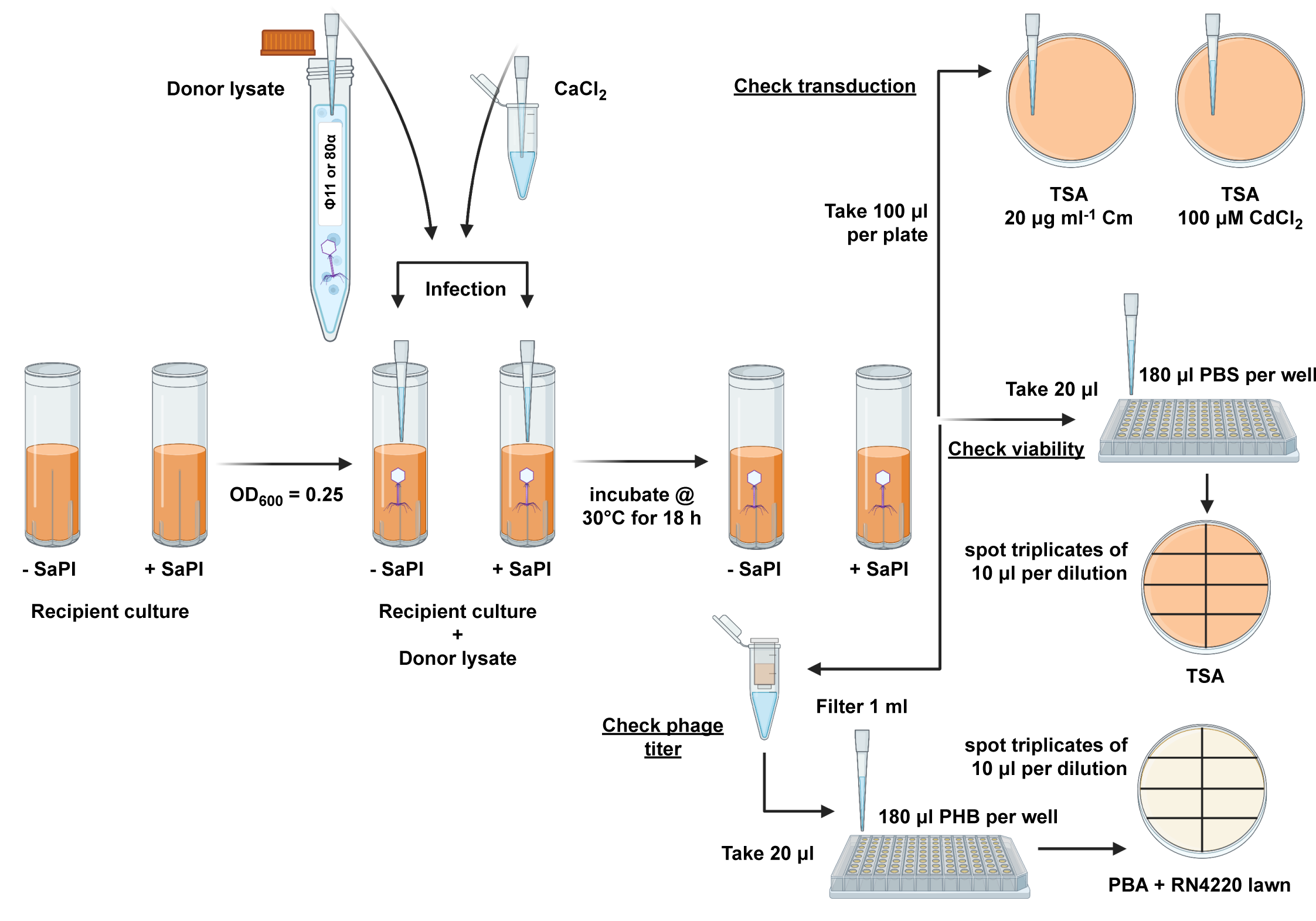

Supplement: S1 Fig — Recipient cultures were grown to exponential phase (OD540 of 0.25, corresponding to ~1 x 10^8^ CFU ml1) in TSB. 5 ml of each recipient culture supplemented with 5 μM CaCl2 were infected with indicated phage lysates at an MOI of 1:10 (phage:bacteria). Cultures were incubated at 30°C and 80 rpm and samplings were performed at the indicated timepoints (1, 4 and 18 h). For transduction assessment, 100 μl of each culture at the defined timepoints were plated directly onto TSA plates supplemented with the appropriate antibiotics. For viability assessment, cultures were serially diluted in PBS and spotted in triplicates of 10 μl per dilution onto TSA plates. For phage titer assessment, 1 ml of each culture was filtered and used for serial dilutions in phage buffer (PHB). Dilutions were spotted in triplicates of 10 μl each onto PBA plates overlaid with a lawn of RN4220. Created with BioRender.com. (TIF) [file pgen.1010146.s001.tif]
