# Supplementary material for: Phage-inducible chromosomal islands promote genetic variability by blocking phage reproduction and protecting transductants from phage lysis
Source: PLoS Genet. 2022 Mar 28;18(3):e1010146. doi: 10.1371/journal.pgen.1010146 (PMC8989297; doi:10.1371/journal.pgen.1010146)
Supplement: Attachment — Submitted filename: Response to reviewers.docx [file pgen.1010146.s002.docx]

Responses to Reviewers' questions

**Comments to the Authors:**

**Please note here if the review is uploaded as an attachment.**

Reviewer #1: Ibarra-Chavez et. al. present measuring the impact of phage-induced SaPI activity on rates of resistance marker acquisition in bacterial recipient cells. They observe increased transduction of resistance markers and decreased lysogeny of inducing phages when bacterial hosts encode SaPIs. From this, the authors conclude that SaPIs actively promote host DNA co-transduction to increase host population genetic diversity and thus increase survival. The work is presented in a concise manner and I appreciated the exploration of the potential importance a non-toxigenic SaPI. Unfortunately, I disagree with the conclusions the authors draw from the data. I find the work to be lacking in novelty and consideration of several major factors that are invalidating to the conclusions in the text (see major points below). I do not recommend publication of this work in any journal without serious reconsideration of the scope of the paper.

We would like to thank the reviewer for the very thorough examination of our manuscript and the supplied comments. We believe to have addressed all the reviewer’s specific comments and useful suggestions to improve the manuscript. However, we strongly disagree with the reviewer’s assessment that “several major factors that are invalidating the conclusions in the text”. Our main conclusion is not that SaPIs are actively promoting host-DNA co-transduction but rather that SaPIs promote the survival of transductants by protecting the overall population and potentially individual SaPI-containing cells from phage predation.

We understand that some the hypotheses that were tested here might seem logical/obvious based on previous literature. However, it is important to remark that no previous papers have validated (or refuted) these hypotheses. Therefore, so far, these remain as unproven hypotheses. This manuscript fixes this problem and provides new insights into the biology of SaPIs/PICIs, and how they impact bacterial evolution.

Major points

1. The manuscript (including the statement in lines 78-79) fails to acknowledge what is currently already known in the broad field of PICI biology regarding PICI-mediated interference with phages. Being familiar with the literature, it is unclear what new biological insights are obtained from this work.

We appreciate the reviewer’s comments and acknowledge that in an attempt for conciseness, some information was not included in the introduction. This has been corrected in the modified version.

However, we completely disagree with the reviewer’s view that no new biological insights were obtained in this work. While it is true that some interference mechanisms have been described in previous literature, their impact on bacterial survival, lysogenization, transduction outcomes and genetic variability of the recipient cells has never been specifically addressed. We therefore strongly believe that our study provides additional biological insights with regards to the impact of PICIs on the acquisition of genetic material via HGT and the generation of genetic diversity in PICI-containing hosts that far surpasses that of PICI-free hosts.

2. Multiple figures in the text do not enrich current understanding of the SaPI system but rather reiterate known information with the tone of novelty.

We disagree with the reviewer’s comment. As previously indicated, while several aspects of SaPI-phage interference have been described in the literature, no study to date has addressed their role in promoting horizontal gene transfer. The data presented here serve to put all these observations into context and are required to understand/explain the impact of SaPIs on transduction, protection from phage killing and the increase in population heterogeneity.

a. Figure 2 shows that phage titer is decreased with SaPI activity. This phage repression activity has previously been explored in molecular detail (Ram et. al. PNAS 2014). The reduction in phage titer is an expected result from what is already known about the system.

We appreciate that SaPIs are known to influence phage titers via a range of interference mechanisms that are expressed after SaPI induction. However, here we study the impact of their interference on the general bacterial population and on the likelihood of transduced cells surviving. While not entirely unexpected, it is nevertheless required to describe the experimental system used in this study (particularly since the combinations and conditions used here do not match those of previous studies).

b. Figure 2 also shows increased cell viability in the presence of SaPIs, which was demonstrated in the original molecular characterization of the system (Ruzin et. al. Mol Micro 2001) and continues to be a theme in SaPI biology. Similarly, Figure 3 shows a decrease in lysogenization frequency in hosts with active SaPIs, another phenomenon described in the original molecular characterization of SaPIs by Ruzin et. al.

See point (a). Importantly, please note that the cited paper does not contain any information on the impact of lysogenization in a SaPI containing strain. An earlier study by Lindsay et. al (1998, Mol Micro), briefly mentioned that there was a higher number of survivors in a bacterial population containing SaPI1 after phage infection compared to a strain lacking SaPI1. However, the number of bacterial cells was nevertheless lower compared to the number of infected bacterial cells (3% survivors in the SaPI-containing strain compared to 0.2% in the SaPI-free strain). In our study, we find that SaPI-containing strains not only survive better than the SaPI-free strain, but also that this population does not undergo phage killing as observed by Lindsay et al. By contrast, the SaPI-containing cells result in an approximately 10-fold increase in viable cells compared to the inoculum while the SaPI-free strains show a 10-fold reduction in viability. The differences in our results to those published previously are likely caused by different experimental conditions and the use of different SaPIs relative to Lindsay et al. Here we study a stabilized system after a prolonged infection and transduction cycle. However, the observed differences were also evident as early as four hours post infection and persisted and increased over time. We have added an additional supplementary figure detailing the relevant changes over time to each of the parameters measured in this study for further reference (Fig. S2).

3. The data and resulting analysis confuse rather than clarify a fairly intuitive concept: if a host cell harbors a phage-interference mechanism like a SaPI, phage infection will be impaired resulting in decreased lysogenization (because fewer phage particles can be produced) and increased host population survival which translates to increased probability of bacterial DNA uptake by transduction. I would argue that any mechanism that impairs phage reproduction in a population could effectively result in the same phenomenon. Thus, there does not appear to be any new biological insights gleaned from this work.

The reviewer highlights the very concept of the proposed manuscript and its aim to verify and provide experimental data to either confirm or refute this hypothesis. In science, ideas must be confirmed or discarded, and this is what we have done here. To provide experimental support and scientific evidence to a logical hypothesis.

While fragmented data has been published in literature addressing aspects of phage – SaPI interference, their impact on horizontal gene transfer has not been specifically investigated. Our study provides this valuable information and confirms the crucial role of SaPIs in promoting HGT and population heterogeneity. In order to make this more evident, we have added new experimental data (where requested) and have substantially modified the figures presented in the manuscript. We believe that with these changes, the intuitive concept should be more accessible to the reader and would like to thank the reviewer for pointing that this was not transmitted in sufficient clarity.

4. The authors draw the conclusion that increased population diversity of recipient cells containing SaPIs acts as an ecological bet-hedging strategy to promote survival in stressful conditions. I do not believe that the data presented in this manuscript directly support this claim. Further assessment of the relative fitness of these populations is required in order to make such a claim about an ecological strategy.

We believe that further assessment for the purpose of this study is not required. HGT can have both positive as well as negative impacts on each individual cell that is transduced with foreign DNA. The outcome will depend primarily on the environmental conditions that the transductants are exposed to. By allowing the generation of a larger pool of heterogeneous bacteria within the population, even if the individual differences might be minute, the overall chances of the bacterial population to contain cells that are better adapted at surviving changing environmental conditions increases. This pressure then automatically leads to the expansion of the best adapted bacterial clone. Thus, by increasing the initial pool of heterogenous bacteria, the overall population fitness is likely increased, and natural selection will promote the expansion of the best adapted clone.

Minor points

1. (Lines 44-48) It is unclear whether all surviving cells in these populations have acquired new DNA.

Not all bacteria that survive have acquired new DNA. As can be seen in Figure 2C&D, the number of viable cells overall is increased in the presence of the SaPI (>10^9^ CFU per ml). By contrast, the number of transductants ranged from 10^3^ to 10^4^ (Figure 1) indicating a relatively minor proportion of the viable population. It is therefore unlikely that the entire bacterial population was transduced with new DNA. We have highlighted this better in the new version of the manuscript.

2. (Lines 54-55) The different types of transduction (generalized vs. specialized vs. lateral) need some clarifying definition in the text.

The introduction has been expanded to accommodate this.

3. Line 87 the designation of the SaPI pT1028 is unclear; this nomenclature is appropriate for a plasmid. If the original publication (is there a citation for this SaPI?) erroneously referred to this element as a plasmid it should be corrected and not perpetuated in the literature

We agree with the reviewer that the nomenclature for SaPI pt1028 is confusing and that many recent publications still refer to it as a phage. This confusion stems from the initial publication describing it as an incomplete phage (Kwan, T., Liu, J., DuBow, M., Gros, P., and Pelletier, J. (2005) The complete genomes and proteomes of 27 *Staphylococcus aureus* bacteriophages. Proc Natl Acad Sci U S A 102, 5174-5179), and which has not been further corrected. The current preferred naming convention for SaPIs indicates the use of SaPI followed by the strain it was isolated from. Thus, in line with this convention, a more appropriate name for the SaPI would be SaPINY940. This has been modified in the text.

4. Lines 89-90 could be clarified by stating here (rather than later) that these phages are the inducing phages for the SaPIs. I would also like some clarification on cross-activity between the selected SaPI-phage pairs (i.e. can phi11 induce pT1028 and 80alpha induce SaPIbov1?)

We agree and have clarified this.

5. A model of the experimental set-up would greatly aid the reader’s understanding of lines 80-101.

We have added a model to the supplementary material (Fig. S1).

6. Consider referring to RN4220 and other strain numbers as something more understandable to the general reader (e.g. “susceptible host”)

We appreciate the reviewer’s suggestion and have modified the text to clarify this point.

7. Please refer to the specific sub-panel of figures referenced in the text (e.g. line 128 should specifically reference Figure 3A)

Done.

8. Line 77 wording requires clarification regarding PICI and host DNA co-transduction.

Clarification added.

9. Line 130 and elsewhere, please detail the actual degree of reduction rather than just calling it statistically significant.

Done.

10. Lines 175-181 need editing, there are words missing from some of these sentences that are required for grammatical correctness and clarity, specifically “creating news that” and “since as mentioned the output”

Thank you for pointing this out, we have corrected this.

11. Figures 1-4: please provide information about the limit of detection for these assays.

The limit of detection for transduction experiments is 10 CFU per ml, 100 CFU per ml for viability and lysogenization titers and 100 PFU per ml for phage titers. Limits have been added to the respective figure legends where required.

12. Figures 1, 2, 3B: One-way ANOVA statistical analysis is not appropriate for these data.

We disagree. While this would be the case if comparisons were only made between the two strains either containing or lacking the SaPI when infected with a single phage, here we are also comparing with the same strains that were infected with a phage mutant. ANOVA was used to identify pairs of groups that show significantly different variance. Significance levels were then calculated using EMMs (Estimated marginal means (EMMs), a.k.a. least-squares means) in R-Studio.

13. Figures 1B, 2B: It appears that there is still some SaPI activation in the absence of sri in 80alpha. This is not addressed in the text.

SaPINY940 appears to have an additional interference mechanism that is independent of SaPI replication. This is currently under investigation in the lab. Nevertheless, SaPI replication is clearly involved in the observed reduction in phage titers, increased viability and increased number of surviving transductants. We have added an additional section in the discussion to highlight this better.

14. Figure 3: Experiments conducted in duplicate are compared to experiments conducted 5 times. This seems inappropriate considering nearly all other experiments were conducted in biological triplicate at minimum.

Thank you for bringing this to our attention. We have performed additional replicates and incorporated them in our analysis. This did not change the interpretation of the presented data.

15. Figure 4: Individual data points on the bar graphs (as in Figures 1-3) are absent on this figure. This figure would also be strengthened by inclusion of a third biological replicate.

We agree and have change this as suggested.

16. Figure 5 is extremely confusing and has many elements that are not factually correct. It could be clarified by removing the shading in the large squares, including the initial phage infection step to orient readers to the overall process, and some serious reconsidering of arrow colors. This figure also contains some misleading information, specifically in the right-side panel that shows an individual SaPI+ cell surviving phage infection (it does not in fact protect the individual cell) and successful transduction blocking further phage infection (also not supported by what is currently known). This figure should be seriously reconsidered.

We disagree with the reviewer’s comments. As mentioned before, not all bacteria that survive have acquired new DNA; however, all the bacterial in the culture were SaPI+. Interestingly, cell viability in SaPI-containing strains never drops below the inoculum suggesting that SaPIs can indeed afford protection to the individual host cell (see new Fig. S2). By contrast, SaPI-free recipients show a reduction in viability. We have expanded our model to incorporate both potential outcomes for the phage after infecting a SaPI-containing strains and have modified the color scheme. We would also like to refer to reviewer 3, who highlighted that our data support our proposed model.

**Reviewer #2:** Interesting manuscript pointing to a central role of PICI's in transduction and importantly also for lysogenization which could be highlighted more and discussed: Why do bacteria avoid becoming lysogens and rather carry PICIs?

We want to thank the reviewer for their feedback and comments to improve the manuscript. We appreciate their interest and recommendations. We believe that spontaneous induction of a lysogenic phage could present a potential threat to the bacteria, therefore harnessing a PICI will enable the host with the protection of acquiring genetic material transduce by the phage particles. Incorporation of a prophage into the bacterial genome also presents a metabolic burden to the organism and frequently prophages are only maintained while providing a benefit to the bacterium. Note that toxins and immune evasion factors encoded in the phage genome are often expressed while in the prophage state. Pathogens often lose certain prophages after jumping to a new host. For example, the *S. aureus* prophage integrated into attachment site 3 is rapidly lost when transferring from human to a bovine or porcine host since the immune evasion cluster carried on the phage is host-specific and provides no benefit in other hosts. PICIs could impose a lower metabolic burden on the host cell relative to a prophage and could thus be favored.

Line 32: A more diverse - more than what? The statement of diversity is somewhat overstating the results of the manuscript. Basically, the diversity is obtained by cells being able to survive without being lysogens - not that a greater diversity of cells in general or transduced cells are being created. The abstract should put more focus on the fact that more cells are able to survive in a phage environment without becoming lysogens.

We appreciate the reviewer’s concern and have added clarification to the abstract. We show that the survival of transductants with antibiotic marker genes is substantially increased. However, we expect this to also true for the transduction of other genetic material. Thus, the survival of transduced populations is highly increased in a recipient harboring a SaPI and thus population diversity is increased substantially. Blocking lysogenization is an additional layer of diversity that can benefit the bacterial population.

line 94: Why is RN4220 being used here? The strain seems in general to suffer from several problems so a strain not having undergone rounds of mutagenesis would be preferable

We appreciate that RN4220 has several deficiencies that render it a less than ideal strain for certain experimental setups. In our experience, however, RN4220 behaves identical to its parental strain RN450 regarding SaPI replication and transduction of DNA. It is used as a universal acceptor in transduction and phage titration experiments widely within the research community. All experiments and their controls have been performed using this background. Thus, we believe that the use of RN4220 is entirely appropriate.

Figure 2, line 368: Are the viable cells transductants or just viable cells in culture?

Most of the viable cells are not transductants for the markers that we tested. Indeed, the highest level of transductants observed is with the SaPI-containing strains and is between 10^3^-10^4^ transductants per ml. By contrast, cell viability ranges from 10^7^-10^9^ for SaPI-free and SaPI-containing strains, respectively (Figure 2). It is possible that a large proportion of cells in the SaPI-containing strain has acquired other foreign DNA unrelated to the antibiotic markers used. However, the majority of cells in the SaPI-containing recipient is likely unmodified.

Figure 3 and figure 4: You are not commenting on the results of figure 3B in the text and basically the data in figures 3 and 4 seem in part to be the same but presented in different ways.

Thank you for the comment. Data in Figure 4 are population frequencies based on the data presented in the rest of the manuscript. While Figure 3 shows the relative proportions observed in a small subset of the bacterial population (the transduced clones). In Figure 4 we aim to expand our view to the whole population of cells in an attempt to quantify the impact of SaPIs in a recipient strain on population diversity. We have expanded the relevant results and discussion sections to clarify this.

line 177: new ones instead of news?

We have revised this sentence.

**Reviewer #3:** The manuscript by Ibarra-Chávez et al, entitled "Phage-inducible chromosomal islands promote genetic variability by blocking phage reproduction" describes a new role for PICIs as promoters of phage-driven gene transfer. The data provided for two different PICIs, induced in the presence of two different phages in Staphylococcus aureus are convincing and overall support the model provided in Fig. 5. The main caveat of the manuscript is that it requires extensive prior knowledge of the PICIs biology and bibliography. The introduction is rather minimalist and does not provide sufficient information to guide the reader toward the initial hypothesis. This version needs improvement to reach the large audience and scope of the journal.

We thank the reviewer for their kind comments. We appreciate the interest and have improved the manuscript to make it more accessible to a wider audience.

L.62: The description and biology of PICIs is rather minimalist, and not really adapted to a large audience. In particular, the phage-encoded inducer needs additional description as it is needed to understand Fig. 1.

Thank you for highlighting this. We have expanded our introduction to give more background information about known PICI-phage interactions.

L. 75, What is meant by “local population?”, if the model considers mixed or non-homogenous populations, it could be further explained.

We have removed the term to avoid confusion.

L.79, it is not clear from the abstract or introduction how the work provided here relates to bacterial (genome) evolution.

We have expanded the abstract to clarify this.

L. 84-88: Two different SAPIs and cognate phages were used in this study for the sake of generalization (as indicated l. 96). Although the use of SapIbov1 is clearly explained, it is hard to get why pT1028 was chosen? A more detailed justification and the addition of references for these two SAPIs models would help the reader. In addition, as the authors study many different PICIs models, why not including different strains and classes of PICIs from different bacterial strains in the study to generalize even more?

The reason why we choose pT1028 (now SaPINY940) as part of our model was because it lacks genes that encode virulence factors, toxins, AMR or biofilm production. Since it has been assumed that the role of PICIs, such as the SaPI family, are exclusively to mobilize and directly transfer beneficial traits to their new host, we wanted to demonstrate that SaPIs can provide additional benefits beyond that. And this is likely why SaPINY940 does not encode any toxins or virulence factors, and simply performs as another phage defense barrier. Here we demonstrate that by defending their host from phage predation, transduction of genetic material enhances. Although, this might well result in bet-hedging, we cannot discard that some of this genetic material could be detrimental and therefore represent a negative impact to the population. We have added this to our discussion.

L.93: Generalized and lateral transduction should be defined in the introduction to understand the subtility of using 2 different reporters.

We agree and have expanded the introduction to incorporate this.

L. 94: Strain RN4220 is a restriction-defective derivative of RN450, as indicated in Table 1. Why such a strain is needed. Table 1 could also mention the genotype of the original RN450.

We appreciate that RN4220 has several deficiencies that render it a less than ideal strain for certain experimental setups. In our experience, however, RN4220 behaves identically to its parental strain RN450 regarding SaPI replication and transduction of DNA. It is used as a universal acceptor in transduction and phage titration experiments widely within the research community. All experiments and their controls have been performed using this background and we expect no differences in RN450. Thus, we believe that the use of RN4220 is entirely appropriate. We have added the original genotype of RN450 into the table.

L.97, is there a reference to provide for pT1028 induction by 80α?

There is no published reference that specifically addresses activation of pt1028. However, this PICI contains a repressor almost identical to SaPI1, and replication of SaPI1 can be induced by the 80α-encoded inducer Sri. Induction of pt1028 (SaPINY940) has been experimentally confirmed within the lab but is subject of another study. We previously reported the combination of a WT and a rebooted pT1028 with 80α in Ibarra-Chavez et al. BioDesign Research 2020 and have added this point to the results section.

L.99, the hypothesis mentioned could be rephrased to help the reader.

Done.

L. 110, it is hard to understand the basis of such hypothesis without additional explanation. If the phage titer decreases, is it because each cell is less productive, or because cells become less susceptible?

This is an interesting point to highlight. In fact, it is currently not known whether each individual SaPI-containing cell is less productive or whether the SaPI affords individual protection against phage infection and lysis of its host. Present studies have always been done at population level and have not followed the fate of individually infected cells. Because release of phage and SaPI progeny depends on the lysis of the host cell, one could argue that either the host cells can become resistant to substantial levels of phage infection or that phage burst sizes are notably reduced. It would be fascinating to follow this up in a future study focusing on individual cell fate rather than the overall population. We have expanded the provided model to incorporate both potential outcomes.

L. 117, the link between lysogeny and transduction facilitation is not obvious as stated.

We have added a sentence and a reference detailing the mechanism in detail to the section for further clarification.

L. 156, in this experiment the genetic variability observed is driven by the co-occurrence, and maintenance in the overall population, of several type of cells. Does it influence the population heterogenicity on the long term? How were the time of plating (18 h post-infection) chosen? In other words, is it possible to obtain a different distribution depending on the incubation time?

This is an interesting observation, and we believe that changing environmental condition will eventually impact on the different phage/transduction dynamics observed in our system. We chose 18 h post infection based on initial time course data where we monitored cell viability, phage and transduction titers at 1, 4 and 18 h post infection (these data have been now included in the manuscript). We observed that transductions occurred rapidly after infection (1 h) and the number of transduced cells increased only incrementally after that when a SaPI was present in the recipient cell. By contrast, the number of transductants was identical in the SaPI-free cells 1 h after infection but reduced significantly over the time course of the experiment. 18 h was chosen as a stabilized final state outcome of the experiment. We acknowledged that population frequencies might be different at the different time points tested but believe that our selected final readout is nevertheless describing the behavior of the experimental system accurately.

Discussion: Could the authors precise why they mention lysogenisation is a stochastic process and provide the appropriate references?

We have included the following references for this process:

Arkin, A., Ross, J. & McAdams, H. H. Stochastic kinetic analysis of developmental pathway bifurcation in phage lambda-infected *Escherichia coli cells*. *Genetics* **149**, 1633-1648 (1998).

Joh, R. I. & Weitz, J. S. To lyse or not to lyse: transient-mediated stochastic fate determination in cells infected by bacteriophages. *PLoS computational biology* **7**, e1002006, doi:10.1371/journal.pcbi.1002006 (2011).

Robb, M. L. & Shahrezaei, V. Stochastic cellular fate decision making by multiple infecting lambda phage. *PLoS One* **9**, e103636, doi:10.1371/journal.pone.0103636 (2014).

As stated in the text, the heterogenicity of the population driven by SAPIs could provide an advantage to the overall population. This could be tested experimentally.

We agree with the reviewer that this could be tested experimentally but we believe this is currently outside the scope of the manuscript. See comments to reviewer 1.

Additional minor points: Phage titer instead of “phage titre”, several occurrences. Strain RN450 is missing in Table 1.

We appreciate this comment. “Titre” is indeed the correct spelling using British English. However, PLoS Genetics being based in the US, we have changed all spelling within the manuscript to American English.
