# Supplementary material for: Phage-inducible chromosomal islands promote genetic variability by blocking phage reproduction and protecting transductants from phage lysis
Source: PLoS Genet. 2022 Mar 28;18(3):e1010146. doi: 10.1371/journal.pgen.1010146 (PMC8989297; doi:10.1371/journal.pgen.1010146)
Supplement: Attachment — Submitted filename: 20220208 Response to reviewers.docx [file pgen.1010146.s003.docx]

**José R Penadés, FRSE**

Professor of Microbiology

Director MRC CMBI

Imperial College London

Flowers Building, Exhibition Road

London, SW7 2AZ, UK

February, 8^th^ 2022

**PLOS Genetics**

Manuscript no: PGENETICS-D-21-00720

Dear Prof. Casadesús,

Thank you very much for providing us with another opportunity to respond to reviewer 1’s comments. In our previous submission, we attempted to respond to the reviewer’s comments intending to provide the required information and experiments that we thought necessary and sufficient to respond to their detailed analysis. Looking at the new and more detailed comments provided in the recent comments, we believe that we might have misinterpreted some of their valid criticisms. We really apologize for that. We have attempted to respond to their comments in detail below and have highlighted where we were uncertain about the precise interpretation of their feedback. We have also formatted the manuscript according to PLOS Genetics requirements. Please let us know if you require elaboration or have any further queries.

Sincerely yours,

**Comments to the Authors:**

**Reviewer #1:**

The manuscript re-submitted by Ibarra-Chavez et. al. includes positive revisions increasing the amount of background information in the introduction and substantial figure revisions that have made the data more aesthetically pleasing. However, the authors failed to address the major concerns cited in the first round of review. Rather than constructively responding to the critiques raised, the authors chose to dismiss nearly every major critique I provided with little to no attempt to address the logic or concern itself. The authors instead chose to re-state their original hypotheses and decline to include any additional experiments or controls that would strengthen the claims they are trying to make. The manuscript continues to fail to acknowledge what is currently already known in other PICI systems where a role in protecting bacteria from phage predation has already been detailed. It is not to say that there are not additional experiments to be done in this area, there clearly are, however if a reader new to this field picked up this paper, they would assume based on how it is written that PICIs interfering with phages has been shown only in this system and here for the first time. Rather than continue to provide detailed feedback on this manuscript I will simply re-iterate a few crucial details that are particularly troubling:

We would like to thank the reviewer for their detailed comments on our manuscript. We had attempted to address all the comments raised by the reviewer in the first round of revision and apologize that we failed in doing that. This was not intended as a dismissal of their criticisms and comments, and we are really sorry if it was seen in that way. Indeed, the reviewer raises several highly valid points that we are happy to include and discuss in the revised version of the manuscript.

However, we would like to point out that we already provided additional data in the revised version of the manuscript (within the supplementary figures) that addressed some of the concerns raised above. We also included references and brief summaries of the known interference mechanism between PICIs and phages in the introduction. Several recent reviews have provided an excellent overview of these systems and we have referred to these for further information. We have expanded this in our revised version of the manuscript to better highlight the current literature and that this is not the first description of the impact of PICIs on phage predation but rather a characterization of how this interplay affects the survival of transductants and overall genetic diversity.

1. The model in figure 5 has undergone edits and is still extremely misleading and unclear. The authors conduct experiments at MOI 1:10 (phage:bacteria) and do not see a change in cell viability in SaPI+ hosts, which they have interpreted here as SaPI+ cells surviving phage infection at the single-cell level. This data simply cannot be interpreted as survival or SaPI-mediated protection at the single-cell level. At the very least a high MOI infection should be performed to test this hypothesis directly and provide actual supporting data. It is also extremely unclear from the model that transduction results from an initial round of phage infection.

We would like to thank the reviewer for this comment and agree that an infection experiment using a high MOI would provide more support for our hypotheses. Following the reviewer’s comments, we have now performed this experiment and included it as new Figure 4. We reasoned that, using a MOI of 1:1, most, if not all, recipient cells would be immediately infected by a phage. In addition, should the observed differences in phage titers and viability persist between the SaPI-containing and the SaPI-free recipient strains, this would support a model whereby SaPIs can afford the individual cell with protection from phage predation (i.e. there is an increased chance of these cells to survive phage infection and not just a reduction in the number of phage progeny produced after infecting SaPI-positive cells). This does not exclude that there is population level protection also and, in fact, this is likely to play an important role as well. However, we believe that this additional data provides evidence for protection at the individual cell level as well. We have modified our model to reflect this.

2. The SaPINY940 data is not explained satisfactorily in the text. SaPINY940 is stated to be induced by sri but the citation for this information (Ref. 26 Tormo-Mas et. al. Nature 2010) does not include this SaPI in its analysis. It is unclear how the authors conclude that Sri is the inducer and unclear if there is SaPI induction in the absence of Sri. From the data in this manuscript, it appears there is some level of induction without the inducer, which makes this SaPI a questionable choice to include in this analysis attempting to connect co-transduction with SaPI activation. If the authors wish to suggest that SaPINY940 has some additional phage resistance capacity outside direct phage parasitism, this should be explored more thoroughly, especially considering the conceptual similarity to recent discoveries in other satellite-encoded defense systems (e.g. Bikard Lab <https://www.biorxiv.org/content/10.1101/2021.01.21.427644v1>). I expect the authors to conclude such investigation is outside of the scope of the current manuscript, I can agree with that provided that the net result of such protection (at the single cell level) is not included in the model (figure 5).

We thank the reviewer for their comments. SaPIs are generally repressed by highly specific repressor proteins and are thereafter induced by the interaction of a phage-moonlighting protein that alleviates this repression. Several different SaPI repressors are common among different SaPIs and SaPINY940 contains a repressor identical to that present in SaPI1. The inducer for SaPI1 is Sri and the Sri encoded in 80alpha is a potent inducer of this island as described in the cited paper (Ref. 26 Tormo-Mas et. al. Nature 2010). As such, SaPINY940 is induced by 80alpha but not by the 80alpha *sri* mutant. In fact, this is very clear when the different phages (wt and *sri*-mutant) are used, in the different experiments. While the first increases transduction of the markers, and reduces phages titers, the *sri* mutant does not do this to the same extent. Note that phage interference only occurs when the SaPIs are induced.

The reviewer is correct in highlighting that the 80alpha *sri* mutant in presence of SaPINY940 maintains some of the phenotypes observed when infecting with the wt phage but at much reduced levels. We agree that this is intriguing. We cannot rule out that this island has additional and uncharacterized mechanisms of phage interference, and we are currently exploring this idea. Our choice of SaPINY940 was based on the lack of evidence for harboring any beneficial traits in its genome, such as virulence or antibiotic resistance genes. Therefore, the fact that this island persists in nature suggest that the results obtained here are relevant in terms of promoting bacterial evolution.

To complement our analysis, and in response to the comments made by the reviewer, we have now included a third PICI, SaPI1, and infected strains devoid (RN4220) or containing the island with either phage 80alpha or its *sri* mutant. Like SaPINY940, SaPI1 is induced by the 80alpha Sri protein, and our experiment clearly showed that presence of this island in a recipient strain also promotes the acquisition of genetic material, reduces phage titers and increases the number of viable cells after infection. However, in the case of SaPI1, these benefits are fully coupled to phage replication.

3. I do not agree that the authors have discovered a novel role for SaPIs (or PICI’s in general) in diversity-generating transduction. The authors do not include controls to convince readers that SaPIs specifically (and not any phage defense in general) can have this effect.

We apologize here because we think we did not explain our thoughts correctly. We did not want to say that only PICIs/SaPIs play a role in promoting diversity. By contrast, we wanted to convey that this is a new and uncharacterized role for the PICIs/SaPIs. In fact, we already mentioned in the previous versions of the manuscript that this “increased diversity” was already observed in other anti-phage systems (i.e., CRISPR-Cas). This has been made clearer in the new version of the manuscript.

4. I disagreed with the author’s conclusion that SaPIs promote host DNA co-transduction to increase host population genetic diversity. The authors responded “our main conclusion is not that SaPIs are actively promoting host DNA co-transduction but rather that SaPIs promote the survival of transductants by protecting the overall population and potentially individual cells from phage predation”. If that is the case the authors should more carefully state their claims, for example, the authors have chosen to title Figure 1 “SaPIs promote increased levels of horizontal DNA transfer.” These details are in direct conflict with the statement in the authors’ rebuttal.

We apologize for these inaccuracies. We have edited the manuscript to make this clear throughout the document.

5. A note about statistics: I am not questioning general significance of the differences being claimed, the averages are obviously different by eye. However, ANOVA analysis with post hoc EMM is not the most appropriate method for analyzing these data sets. Every comparison made in this manuscript is between 2 well-defined groups that do not appear to have similar distributions. This type of data is best analyzed by Student t-test, which does not assume that your data are distributed the same in all sets. ANOVA assumes all sets have equal distribution and attempts to find relevant pairs of groups to analyze. The experimental set-up here defines these groups so this is not necessary. A single t-test per group results in the lowest probability of making a Type I error. Choosing ANOVA and then post hoc analysis may “work”, but it increases the risk of Type 1 error, artificially inflates the p-value, and is an unnecessarily complicated analysis for the type of data being presented in this manuscript.

We would like the reviewer for taking the time to explain their rational for the statistical analysis. This has been extremely helpful, and we fully appreciate the time the reviewer has dedicated to this. We have improved our statistical analysis pipeline and included more test to analyze the distributions, etc. of our datasets to select the analysis more appropriately in the future. As the reviewer clearly has highlighted, the comparisons are made between two groups only and no further comparisons are required to assess our key hypotheses. We have now corrected our statistical analysis performing t-tests. This has retained the overall statistical relevance of our results.

Reviewer #2: I am happy with the response

We would like to thank the reviewer for the comments.

Reviewer #3: Overall, the text of the manuscript has improved.

We would like to thank the reviewer for the comments.

Title could be completed: Phage-inducible chromosomal islands promote genetic variability by blocking phage reproduction and protecting the transductants from phage lysis

We agree and have changed the title.

Line 41: “of the general bacterial population”, this is not obvious what is meant by general population, at the population level ?

We have changed this to “the overall survival of the bacterial population as well as the transductant cells”.
