# Supplementary material for: Phage-inducible chromosomal islands promote genetic variability by blocking phage reproduction and protecting transductants from phage lysis
Source: PLoS Genet. 2022 Mar 28;18(3):e1010146. doi: 10.1371/journal.pgen.1010146 (PMC8989297; doi:10.1371/journal.pgen.1010146)
Supplement: Attachment — Submitted filename: Response to reviewers.docx [file pgen.1010146.s004.docx]

**José R Penadés, FRSE**

Professor of Microbiology

Director MRC CMBI

Imperial College London

Flowers Building, Exhibition Road

London, SW7 2AZ, UK

March, 10^th^ 2022

**PLOS Genetics**

Manuscript no: PGENETICS-D-21-00720

Dear Prof. Casadesús,

Thank you very much for providing us with another opportunity to respond to reviewer 1’s comments. We have attempted to respond to their comments in detail. We have also formatted the manuscript according to PLOS Genetics requirements. Please let us know if you require elaboration or have any further queries.

Sincerely yours,

**Comments to the Authors:**

**Reviewer #1**: The manuscript is much improved from the initial submission, but there are some points on which it appears we will have to agree to disagree. The experiments that were used to conclude that "SaPIs protect individual cells from phage attack" are inappropriate and do not make any sense to me. At an MOI=1, ~63% of cells are expected to be infected, unless the authors have a compelling reason to indicate the Poisson distribution does not apply to the system under study. The authors state: "We reasoned that if SaPI-mediated protection acted at a population level alone, we should not observe any differences in cell viability, phage, or transduction titers between SaPI-positive and SaPI-negative recipient cells under these conditions. " I disagree. Seeing as nearly 40% of the cells are uninfected, and phage titers are reduced following an initial round of infection by the SaPI it is no wonder the authors have a difference in cell viability. This does not support the model that SaPIs protect individual cells from phage attack. At an MOI =5, 99% of cells would be infected and I am certain that the authors would find absolutely no difference in cell survival +/- SaPI, but that is not the experiment they chose to perform.

Unfortunately, we were unable to test higher MOIs in our experiments as this increased lysogenization of the temperate phages used in this study, which can mask the protection afforded by SaPIs. As the reviewer states, and assuming a Poisson distribution, at an MOI of 1 ~63% of cells would be infected by at least a single phage particle (~37% with one, ~18% with two, ~6% with three phage particles). Around 37% would therefore not be infected. While increasing the MOI to 5 would increase the overall number of cells that are infected with at least a single phage to ~99%, it also increases the number of cells infected by multiple phages and only a minor proportion of cells would be infected by a single phage (~3%). Temperate bacteriophages can incorporate into the chromosome of the infected cell to form lysogens and this is strongly correlated to the number of infecting particles per cell. In P22 an MOI of 5 results in more than 90% of cells becoming lysogenic (MOI 1: 23% lysogens, MOI 3: 45% lysogens) (Steinberg, B. M. and M. Gough (1976). Nature 263(5572): 54-56) and similar observations have been made in phage lambda Kourilsky, P. (1973). Molecular & General Genetics 122(2): 183-195) indicating that in this context infection with 2 or more phage particles will almost certainly produce a lysogen. Both phages in our experimental system are temperate and we would expect similar, if not completely identical, trends to be upheld. At an MOI of 5, 96% of bacteria would be infected with multiple phage particles and the resulting lysogenization rates would likely prevent us from drawing any conclusions as to SaPI-mediated single cell protection. Addressing these points to a satisfactory degree, while highly intriguing, is beyond the scope of the presented manuscript.

In any case, we would like to thank the reviewer for their valid comment. While our major claim that SaPIs promote the transduction/survival of transductants and thereby population heterogeneity remains unaffected by the reviewer’s argument, we fully agree with their argument that our present data do not yet support that individualized cell protection is afforded by SaPIs. We believe that SaPI-mediated individual cell protection is an intriguing possibility, which is likely system-specific and that this should be addressed in depth in a future study. However, we have modified the text throughout to state that the observed behavior could be explained by SaPI-mediated population level protection.

The paragraph from line 209 has been modified as follows:

“SaPIs protect bacterial populations through a range of MOIs.

To test whether SaPIs could mediate population protection when exposed to higher initial phage burdens, we repeated the infection experiment using an MOI of 1:1 rather than the MOI of 1:10 that was used in the previous experiments (Fig 4). This high MOI was selected to ensure that most bacterial cells would undergo a phage infection (~63% at an MOI of 1:1 compared to ~9.5% at an MOI of 1:10 assuming Poisson distribution of infection) as well as to balance the drive of temperate bacteriophages to lysogenize at higher MOIs [34, 35]. Even when cells were infected with 10-fold the number of phages, SaPI-positive cells retained an increased number of transductants, a reduced phage titer and increased viability compared to their SaPI-negative relative (Fig 4). These differences where more robustly maintained in experimental systems involving phage 80α compared to the system with Φ11, indicating that the afforded protection was likely dependent on the phage-SaPI pair as well as the infectious dose.”

We have also modified the discussion to reflect this change (lines 278-290):

“Here we show that PICIs are not merely selfish MGEs but provide additional benefits to their host cells by blocking phage reproduction and thus promoting the survival of the entire bacterial population, including those that have acquired foreign genetic material (Fig 6). While prophages and CRISPR-Cas systems provide defense at the single cell level, PICIs are likely operating at population level through a general reduction in phage titers. While PICI-containing cells show no differences in phage infection, phage burst sizes are known to be reduced in PICI-containing cells that are unsuccessful in fully blocking the infecting phage [19]. Whether PICIs can also contribute to protecting individual cells from phage predation remains to be determined but is likely to be dependent on the individual phage-PICI combination. Untangling the protective effects that are eventually attributable to PICIs or simply the result of lysogenic protection (particularly at high MOIs), could provide further insights into the evolutionary impacts of these versatile molecular parasites.”
